# Supplementary material for: Noise enhances odor source localization
Source: ArXiv. 2026 Jan 12:arXiv:2601.07445v1. Preprint. [Version 1] (PMC12869396)
Supplement: Supplement 1 [file NIHPP2601.07445v1-supplement-1.pdf]

Supporting Information

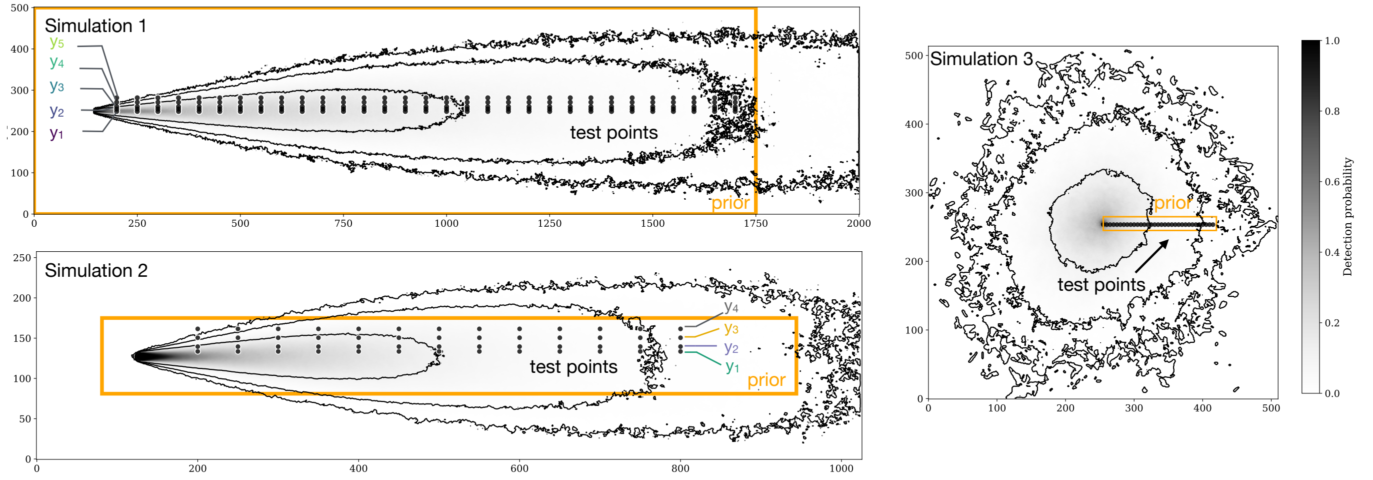

Figure S1. Test locations for simulation 1, 2 and 3. Simulation 1: $y = 0, 5, 10, 15, 20$; prior: 1750×500. Simulation 2: $y = 0, 5, 10, 15, 20$; prior: 863×94. Simulation 3: test points positioned radially in 1D; length of prior: 165. In all panels, increasingly wide contours of the likelihood (black) corresponding to $\ell = 0.1$, 0.01, and 0.001 and all priors are flat within the region indicated in orange.

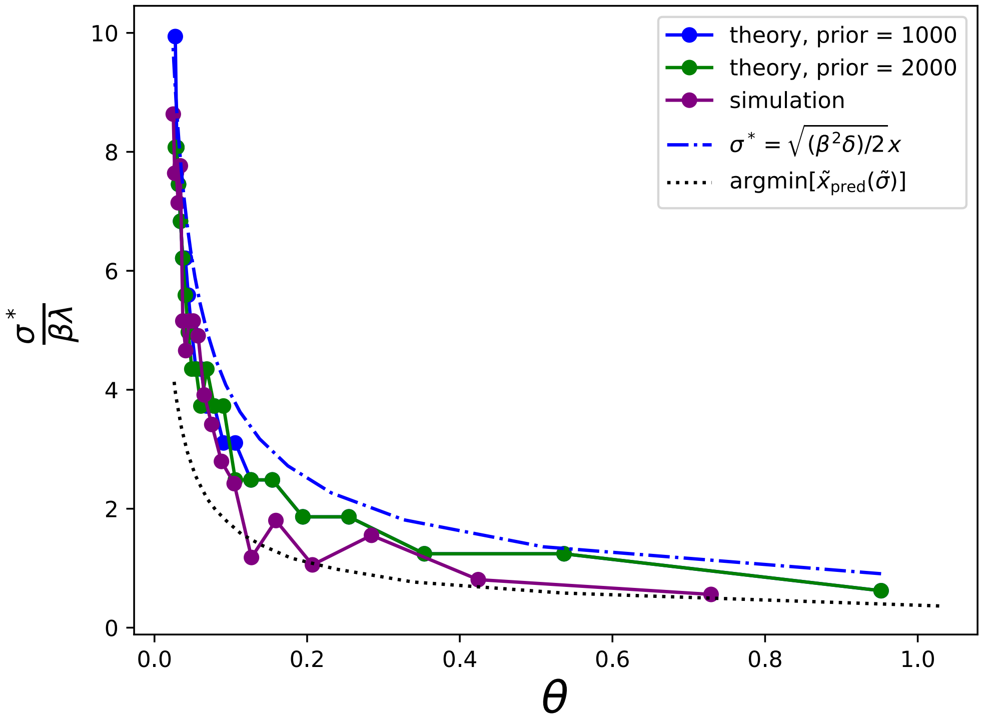

Figure S2. Theoretically optimal size of the multisensor agent, σ^*, normalized with the parameters obtained by fitting the empirical likelihood with the analytic form (eq. 8 main text), as a function of the fraction of detections in the $N \to \infty$ limit, where $\theta = \ell(x, 0)$.

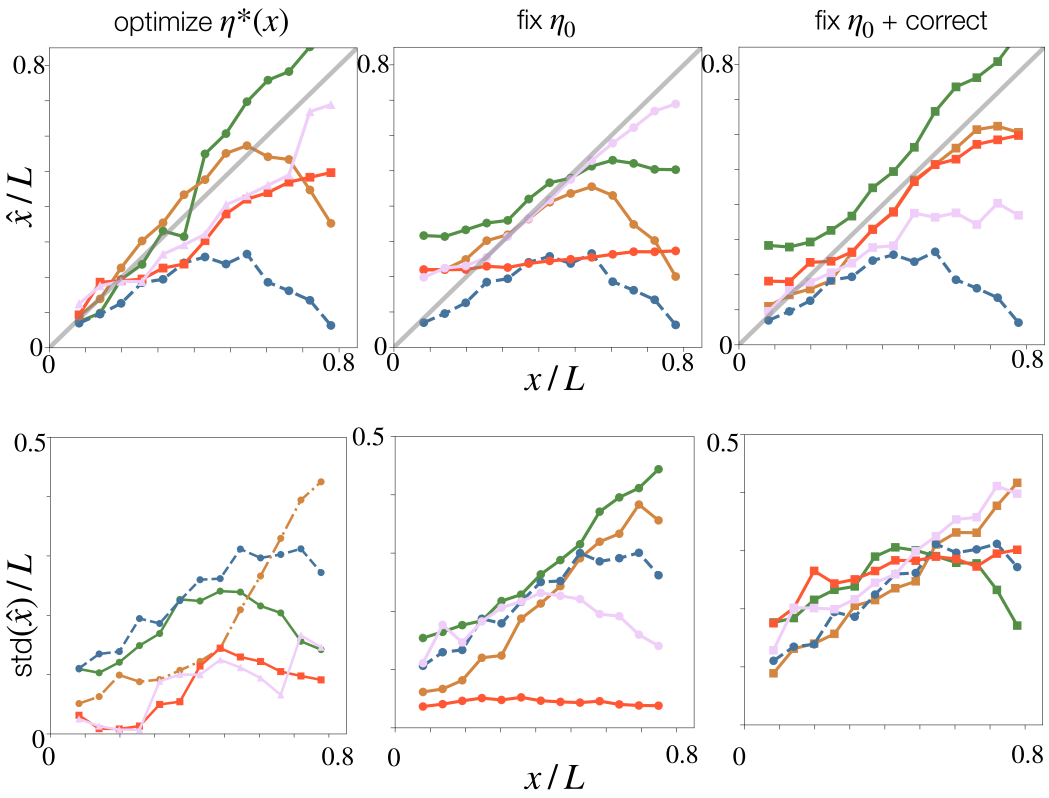

Figure S3. Estimated position $\hat{x}$ (top) and standard deviation std$(\hat{x})$ (bottom) of the source *vs* ground truth x. All quantities are normalized with the length of the prior, L. Different colors correspond to different sources of noise (Color code as in Fig. S4; blue = perfect proprioception) and different columns correspond to different ways of tuning the error: left, noise is tuned to its optimal value; center: noise is fixed; right: noise is fixed and error correction is applied to the inference.

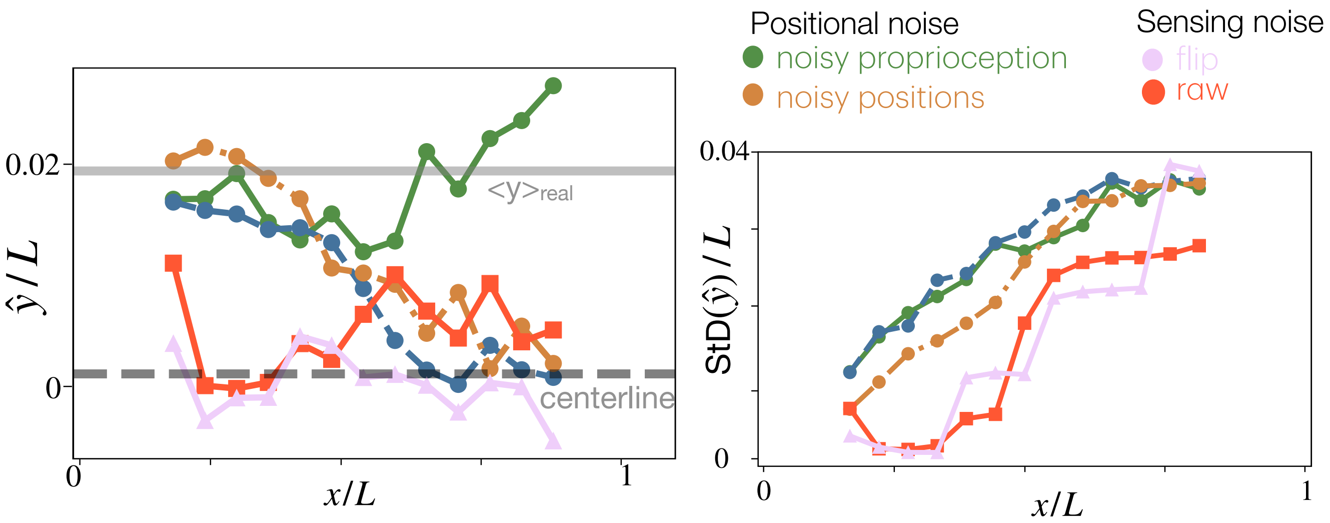

Figure S4. Estimated position $\hat{y}$ (left) and standard deviation std$(\hat{y})$ (right) of the source *vs* ground truth x. For simplicity, we averaged the y prediction across all four values. The gray line represents the correct prediction of the average y, and is marked with $< y >_{real} = (y_1 + y_2 + y_3 + y_4)/4$. All quantities are normalized with the length of the prior, L. Different colors correspond to different sources of noise according to the legend, with noise tuned to the optimal value, defined as the one that maximizes accuracy in the x direction. For positional noise, optimizing inference in x still allows to infer y similar to perfect proprioception (blue curve). Sensing noise, optimized for inference in x, precludes inference in y.

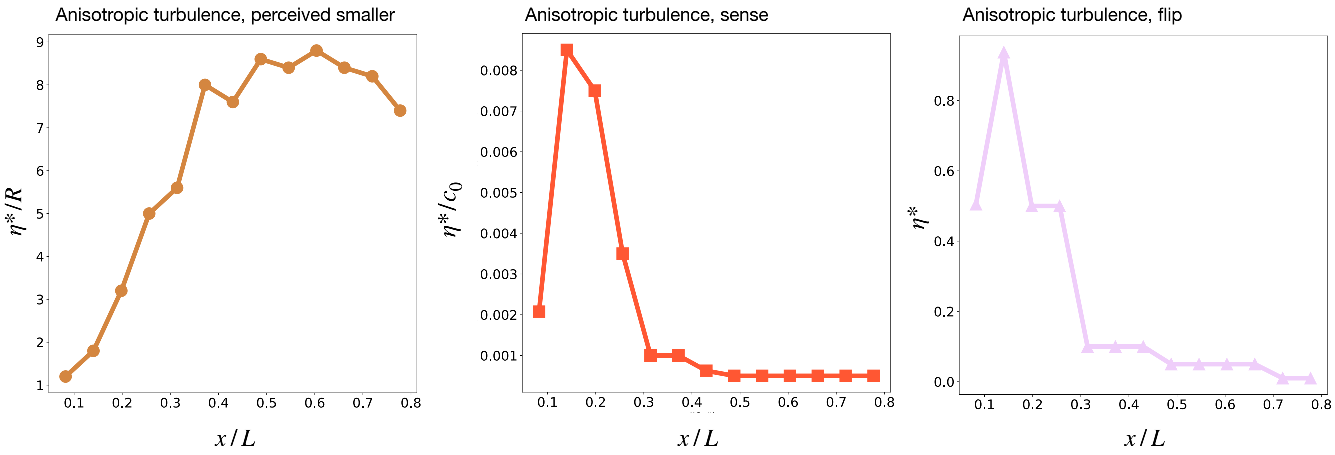

Figure S5. Normalized optimal errors, as a function of distance of the test point from the origin, for positional error on the actual sensor position (left), sensing error affecting the concentration (center) and flipping error, affecting the probability to detect (right).

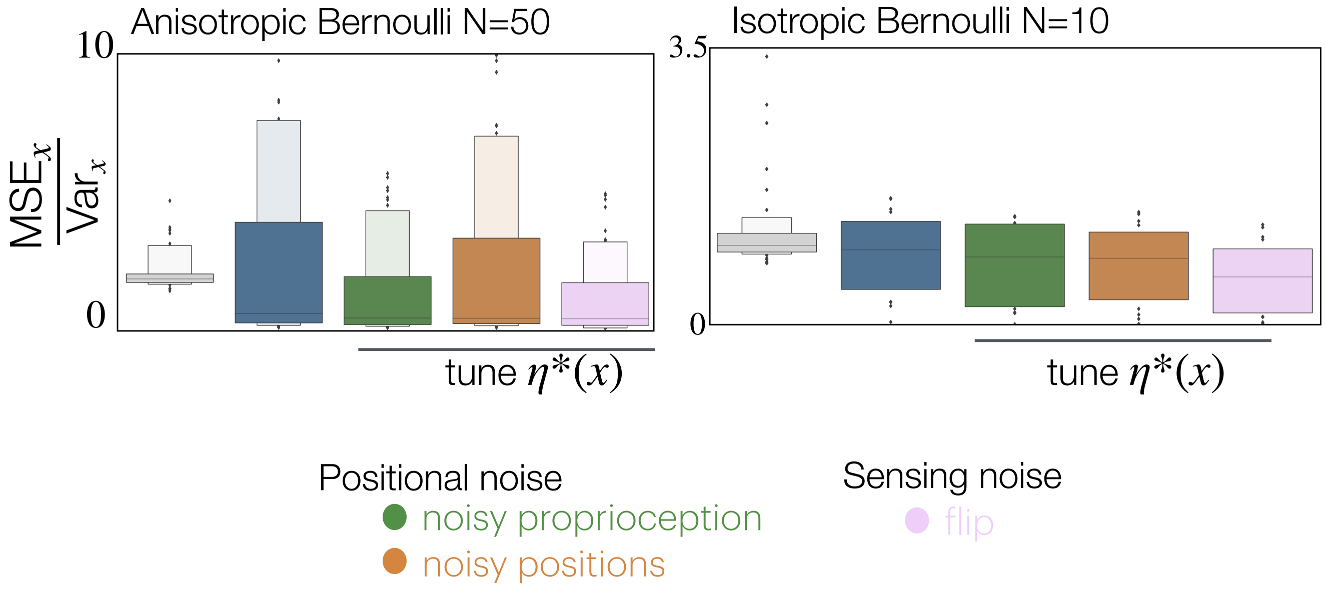

Figure S6. Aggregate statistics on accuracy of the inference, with different noise sources for anisotropic Bernoulli detections with $N = 50$ sensors (left) and isotropic Bernoulli detections with $N = 10$ (right). The number of sensors was chosen so as to make the comparison meaningful, i.e. so that inference with perfect sensing and positional information is inaccurate enough so that it leaves some margin of improvement.

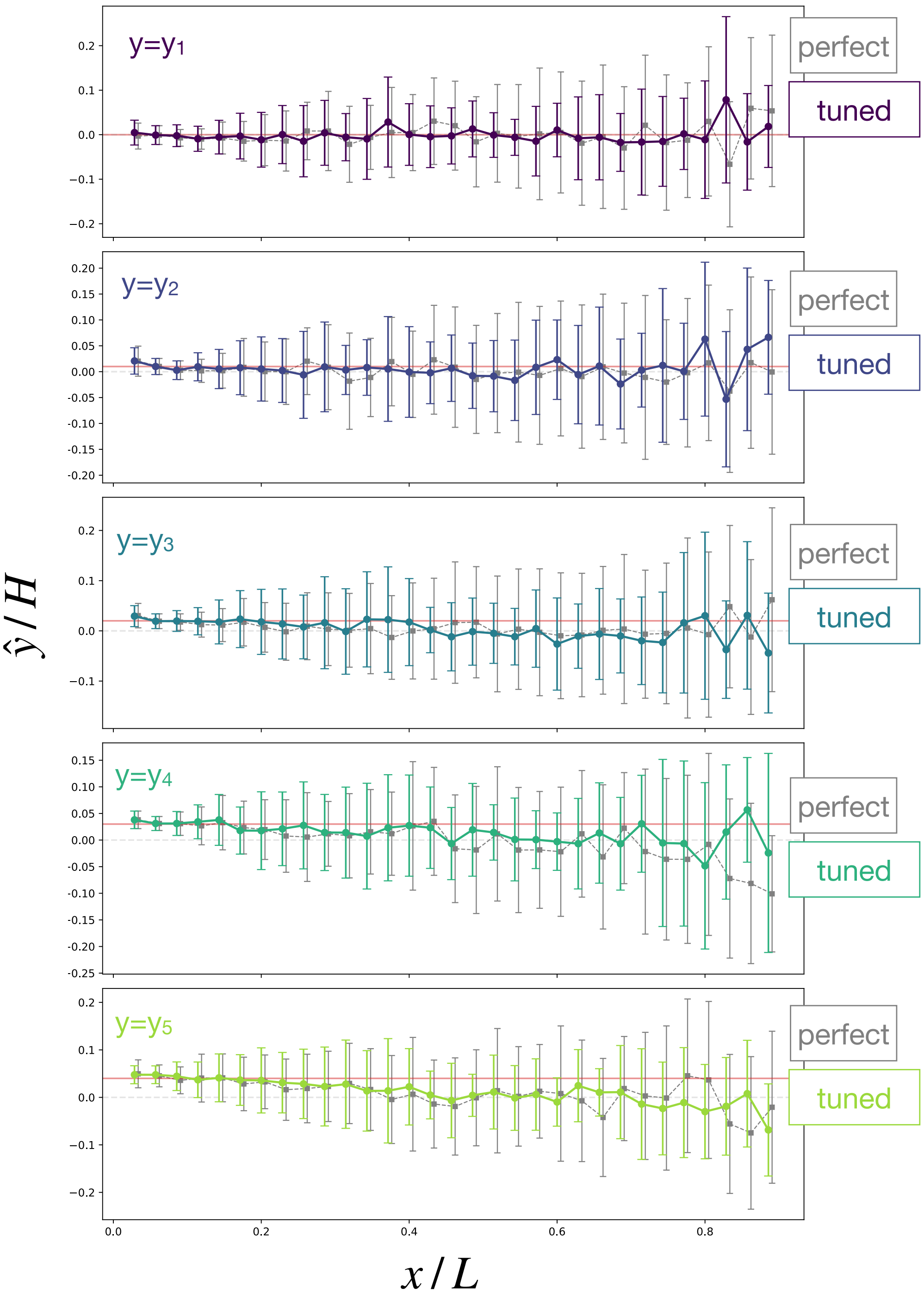

Figure S7. Predicted $\hat{y}/H$ for perfect proprioception as a function of x/L (gray) and optimally tuned noisy proprioception (colors), showing that noise in proprioception does not degrade predictions in y. Red line corresponds to correct y/H; top to bottom correspond to the five values of y indicated in Fig. S1, top left (same color code). Here, L is the length of the prior and the width of the prior is $H \approx 0.3\,L$.

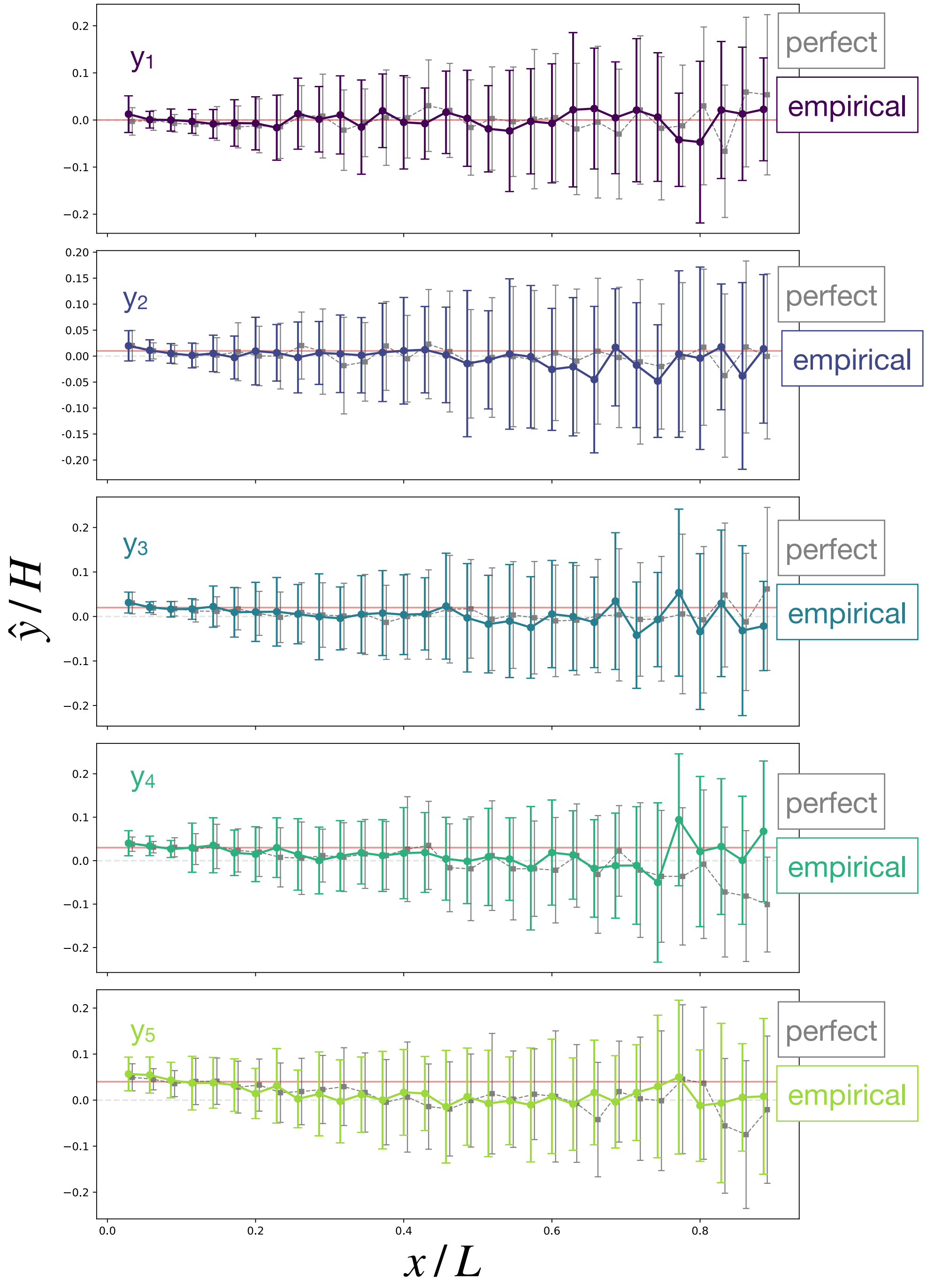

Figure S8. Same as Fig. S7, but with noise tuned empirically.

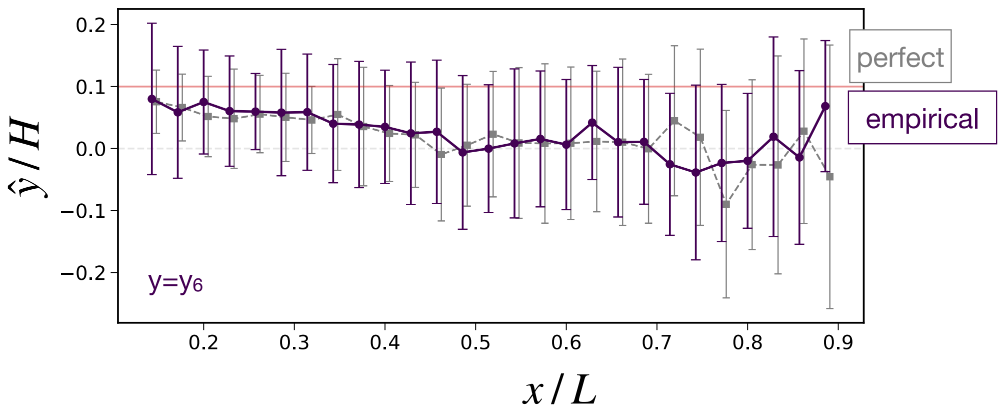

Figure S9. Same as fig. S8, for one additional set of points further from the centerline, $y_6/H = 0.1$, showing similar qualitative results, with degraded inference near the source.

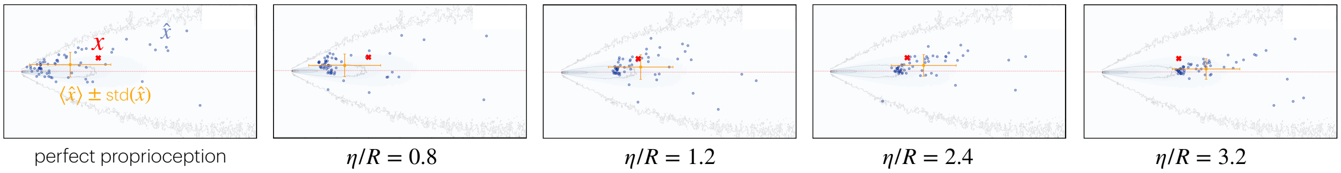

Figure S10. Inferred locations of the test point using noisy proprioception with η set to the value marked under the plot (scatter blue circles); their mean and standard deviation (orange dot and errorbars); real position of the test point (red). From left to right: increasing values of proprioceptive noise move the cloud of inferred positions further away from the source.
